# Pediatric Flatfoot: Is There a Need for Surgical Referral?

**DOI:** 10.3390/jcm12113809

**Published:** 2023-06-01

**Authors:** Manuel Vergillos Luna, Adyb-Adrian Khal, Kara A. Milliken, Federico Solla, Virginie Rampal

**Affiliations:** 1 Department of Orthopedics, Regina Montis Regalis Hospital, 12084 Mondovì, Italy; m.vergillosluna@gmail.com; 2 Department of Orthopedics, Lenval University Children’s Hospital, 06200 Nice, France; adyb_khal@yahoo.com (A.-A.K.); kmillik1@jhu.edu (K.A.M.); federico.solla@hpu.lenval.com (F.S.); 3 Laboratoire Motricité Humaine Expertise Sport Santé, Unité de Formation et Recherche Sciences et Techniques des Activités Physiques et Sportives, 06205 Nice, France

**Keywords:** pediatric flatfoot, pediatric orthopedics, surgical management, foot pain, foot disorders

## Abstract

Pediatric foot deformities are a common finding, concerning up to 44% of preschool aged children. The absence of accepted international guidelines, as well as heterogeneity in definitions and measurements, makes management of pediatric flatfoot a challenge, and decisions surrounding specialized care referral confusing and biased. The objective of this narrative review is to provide guidance to primary care physicians treating these patients. A non-systematic review of the literature regarding the development, etiology, and clinical and radiographic assessment of flatfeet using the PubMed and Cochrane Library databases was performed. The exclusion criteria for the review were adult populations, papers detailing the outcome of a specific surgical procedure, and publications prior to 2001. The included articles showed great heterogeneity in definition and proposed management, which makes the study of pediatric flatfoot challenging. Flatfoot is a common finding in children under 10 years old, and should not be considered pathological unless stiffness or functional limitation are present. Surgical referral should be reserved to children with stiff or painful flatfoot, while simple observation is indicated for flexible, asymptomatic flatfeet.

## 1. Introduction

Pediatric foot abnormalities often push parents to seek specialized orthopedic advice out of fear of permanent foot deformation or disability [1]. Among all pediatric foot concerns, flatfoot is especially frequent due to the hidden plantar arch characteristic of newborns. Prevalence of flatfoot in the preschool aged child has been found to be as high as 44% [2].

Although flatfoot has been defined as a decrease or absence of the medial longitudinal arch (MLA) of the foot, no formal consensus exists as to how low of an arch should be considered physiological. Thus, many authors refer to flatfeet as “normal anatomic variants” [3], as long as no disability or pain is present. Furthermore, variance in the assessment of the MLA during a static or a dynamic position has been shown to change the result significantly, with muscle activity during gait resulting in an increased chance of finding a normal MLA [4].

The absence of accepted international guidelines, along with heterogeneity in definitions and measurement methods, makes management of pediatric flatfoot a challenge, and decisions surrounding referral for specialized care confusing and biased. In many Western European countries (e.g., France, Italy, and Spain), many young children with flatfoot are referred to the orthopedist surgeon because of high parental restlessness and insistence. Concerns regarding the feet are even said to be the main reason for a visit to the pediatric orthopedic outpatient clinic in some countries [5,6]. Moreover, the readability, understandability, and quality of patient education materials about flatfeet on the Internet—which is where parents get most of their information—vary and are often worse than professional recommendations [5].

The objective of this article is to present the authors’ opinion based on their clinical experience and the synthesis of recent literature, in order to provide guidance to primary care physicians and podiatrists managing these patients.

## 2. Sources Selection

A non-systematic review of the literature regarding the development, etiology and clinical and radiographic assessment of flatfeet using the PubMed and Cochrane Library databases was performed on December 2021, then updated on May 2023 using “pediatric flatfoot”, “pes planus”, “plantar arches”, “asymptomatic flatfoot” and “symptomatic flatfoot” as search terms, concerning articles published since 2001. Searches were limited to English language studies. Papers including adults or focusing primarily on treatment results were excluded. The SANRA Guidelines [5] for the quality assessment of narrative review articles were followed to ensure the scientific validity of this research. 

**Figure 1:**
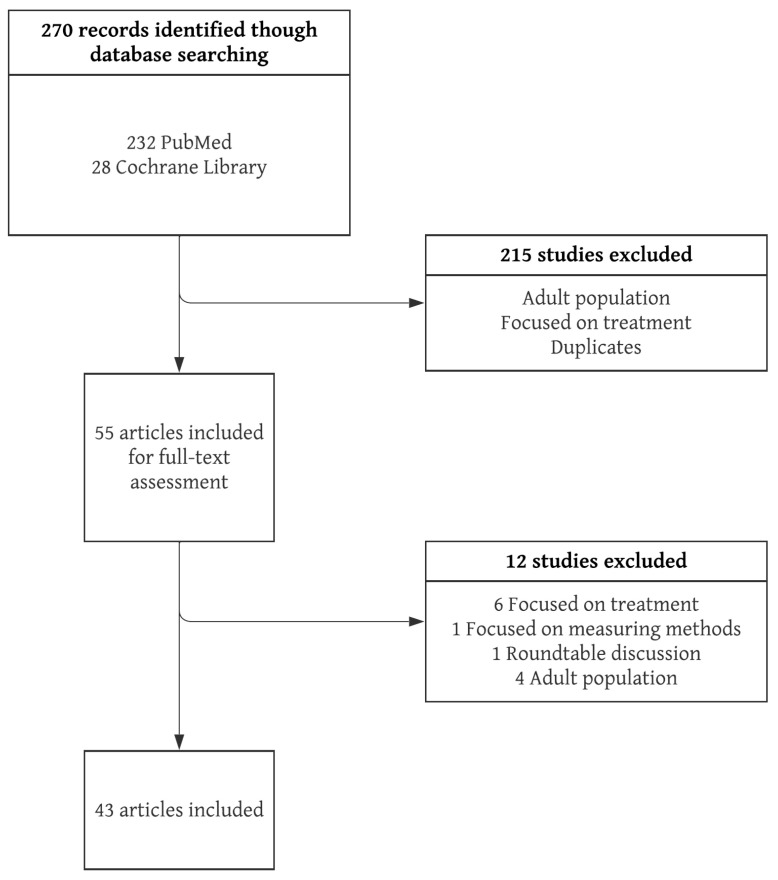
Flowchart of search strategy.

The initial search yielded 270 original papers, of which 54 articles were selected for full-text assessment after applying exclusion criteria and the removal of duplicates. Twelve studies did not pass full-text screening; therefore, 43 original articles were ultimately included in this review (Figure 1).

## 3. Plantar Arch Development

The sole of the foot consists of three plantar arches that support and aid in weight distribution and propulsion: the MLA, the lateral longitudinal arch, and the transverse anterior arch. The MLA is formed by the first three metatarsals, three cuneiforms, the navicular bone, the talus, and the calcaneus bones. The peak of the medial arch is the superior articular surface of the talus. The arch itself is supported by numerous ligamentous and muscular structures, which allow for the lengthening and shortening of the arch during the gait cycle.

**Figure 2:**
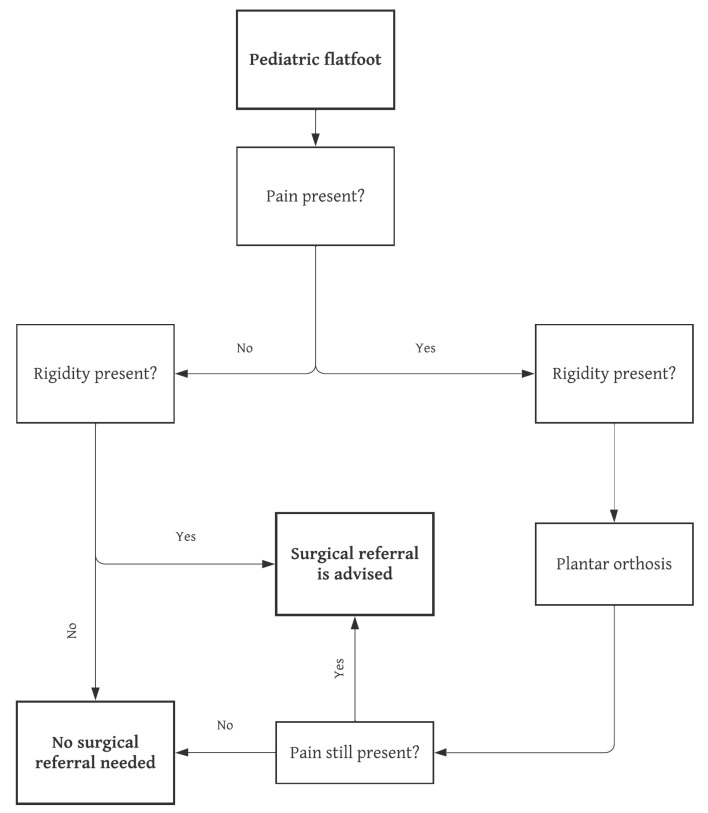
Recommended management of pediatric flatfoot.

Typically, newborns present with flatfeet due to persistence of medial fat pads and undeveloped longitudinal arches, which do not appear for 4–5 years. Afterwards, stiffness of the bony and ligamentous structures, along with strengthening of intrinsic and extrinsic muscular components, will help structuring and maintaining the MLA during orthostasis [7,8]. Maturation of the arches continues after the preschool age, and numerous studies have confirmed that, even though critical changes of the foot occur frequently before the age of 6, “normal” clinical and radiographic values will be achieved no sooner than 9 or 10 years of age [7,8,9,10]. Moreover, further maturation may be seen during adolescence [11]. Specialized consultation should be avoided for flexible flatfoot before the age of 5, given that mild plantar arches represent a normal developmental phase. Controversy exists regarding management of flatfoot in older children; however, common practice dictates that specialized advice is of little use unless symptoms such as pain or fatigability appear (Figure 2).

## 4. Etiologic Classification

Pediatric flatfoot is mainly classified as flexible flatfoot (FFF) or rigid flatfoot (RFF). FFF is characterized by a normal MLA while non-weight-bearing or with external maneuvers that disappear when standing. Children with RFF present with persistent low or absent MLA. FFF has been associated with male gender, increased body mass index, and shorter body height for all grades of severity [12]. Although no high-quality evidence exists focused on pain incidence in untreated FFF, the majority of FFF rarely causes concern. Nonetheless, conservative or surgical intervention may be justified in the case of a short Achilles tendon and/or pain [13,14,15]. 

On the other hand, specialized surgical advice should be sought for all RFF cases in order to rule out congenital vertical talus (CVT), tarsal coalitions, and neuromuscular conditions. CVT associates a severe equinus of the rearfoot to a rigid rocker-bottom appearance. If pathology passes misdiagnosed until walking age, symptoms may include difficulty with weight bearing and wearing shoes [12,16]. Clinical examination shows hindfoot equinus, hindfoot valgus, forefoot abduction, and forefoot dorsiflexion in the newborn. Diagnosis will be confirmed by radiographic evaluation if a fixed vertical position of the talus and dorsal dislocation of the navicular bone on the talus is shown [17,18].

Tarsal coalition is the abnormal fusion of two or more bones. Average age of onset is 8–12 years, and prevalence is 2% [12,19]. This union may be composed of bone, cartilage, fibrous tissue or a combination of those. Talo–calcaneal and calcaneo–navicular coalitions make up 90% of tarsal fusions [19]. Most tarsal coalitions are initially asymptomatic, with onset of stiffness and pain after minor trauma or activity change and a history of frequent ankle sprains due to reduced subtalar joint mobility [16,20].

## 5. Symptomatic Criteria

A recent Spanish cross-sectional observational study [21] of 835 adults found a flatfoot prevalence of 27% among the general population, and found a direct correlation between the presence of flatfoot and lower quality of life as well as lower foot function scores.

Static analysis of a pediatric population has shown that increased forefoot abduction could be correlated to the apparition of symptoms [22]. A recent study, however, comparing FFF and typically developing feet (TDF) in children during gait, found reduced ankle and subtalar dorsiflexion with increased forefoot supination and abduction in both symptomatic and asymptomatic FFF, as opposed to TDF [23]. Contrary to previous static analysis, no differences in foot kinematics were found between symptomatic and asymptomatic FFF. Moreover, on gait analysis, differences in ankle kinematics between asymptomatic and symptomatic FFF indicated that symptoms were dependent on tissue wear and subjective pain threshold rather than structural and functional differences.

Although some authors claim that persistence of non-symptomatic flatfoot will lead to tissue overcompensation and ultimately to arthritis and joint destruction [24], there is a lack of high-quality evidence to support this statement. Most clinicians would agree that painless FFF has a low probability of evolving into chronically painful feet after growth has ceased; therefore, no sufficient evidence exists to support “prophylactic” treatment of pain-free FFF in children [25] (Figure 2). The following treatments are described: rest, activity modification, ice, and non-steroidal anti-inflammatories [24,25]. Generic orthotic use has been demonstrated to relieve symptoms in painful FFF, and should therefore be advised in these patients [25,26]. Conversely, their use in asymptomatic FFF is not supported by evidence. 

## 6. Radiographic Assessment

Plain radiographic assessment is frequently utilized as a first approach during the initial diagnostic workup of a suspected flatfoot, even though the clinical relevance of these measurements is unclear or outright unnecessary in cases of asymptomatic feet [27]. The most common radiographic quantitative parameters are summarized in Table 1.

A 2018 systematic review of the literature [28], which included over 15,000 children, studied different clinical and radiological methods for flatfoot assessment. Based on this review, only three clinical measures had enough evidence to support their use within the pediatric population: the Chippaux–Smark index, the Staheli arch index, and the FPI-6. This finding was recently further supported by a Level II prospective study [29]. No radiological measure included in the review demonstrated enough reliability or validity for assessment of the pediatric foot. This included calcaneal pitch, dorsoplantar talocalcaneal angle, plantarflexion of talus, lateral talocalcaneal angle, calcaneal-first metatarsal angle, and talus-first metatarsal angle.

Some correlation has been found between symptomatic FFF and severity of talar inclination measured as talus-first metatarsal angle in a cohort of young adult males [30], but the study limitations call into question its clinical application. Although radiographic examination has yet to prove itself useful in the diagnostic work-up of FFF, weight-bearing dorsoplantar and lateral radiographs are sometimes prescribed because of parents’ repeated demands. Therefore, it has been shown that radiographs could help localize the origin of the deformation. In cases where the foot becomes painful and requires surgery, radiographic evaluation is required to guide surgical intervention, as shown by Bourdet et al. [31]. Comparatively, radiographic assessment of RFF is mandatory, as this often visualizes underlying pathologies, which can include CVT and tarsal coalitions, for which first and/or second level radiographic evaluation is necessary [20,32,33].

## 7. Rare Differential Diagnosis of Flatfoot

### 7.1. Peroneal Spasm

Though peroneal spasm has often been associated with tarsal coalitions, it is possible to observe its association with other conditions, such as traumatic injury or ligamentous strain. Controversies exist regarding its classification and etiology, as EMG studies have demonstrated no muscular spasm, but rather an organic shortening of the muscles [18,33,34].

### 7.2. Neuromuscular Conditions

Disorders affecting the nerves or muscles supporting the MLA may cause flatfeet [35]. Although rare, pes planus can occur due to spasticity of the gastrocnemius-soleus complex [36] or weakness of supporting muscular structures [37]. Neurological examination should be part of the initial flatfoot assessment to rule out such diagnoses.

## 8. Conclusions

The heterogeneity of current definitions and management pose challenges to the study of pediatric flatfoot. No article to date has provided evidence to justify the treatment of flexible, asymptomatic flatfeet; therefore, caution should be exercised when deciding to treat this condition. Plantar orthosis, sometimes combined with foot exercises, is debatable as an efficient mechanism to improve the plantar arch [38,39,40,41,42,43,44]; however, this can relieve medial arch pain associated with flatfoot [40,41]. 

Flatfoot is a common finding in children under 10 years old, and is, to an extent, normal during development. Therefore, pediatric flatfoot should not be considered as pathological unless stiffness or functional limitation are present. Surgical referral should be reserved for children with stiff or painful flatfoot, while simple observation is indicated for asymptomatic feet. A podiatrist referral, if available, should also be proposed as a first step in this context. 

A flowchart based on literature review and the authors’ opinion summarizing the recommended management of pediatric flatfoot can be found in Figure 2 [12,13,14,15,16,17,18,19,24,25,26,38,39,40,41,44].

## Figures and Tables

**Table 1:** Normal values of commonly used quantitative parameters in the assessment of pediatric flatfoot on weight-bearing radiographs.

Projection	Parameter	Normal Value
Dorsoplantar view	Talo-navicular coverage angle	<7°
	Talus-first metatarsal angle	<5°
	Talocalcaneal angle	25–40°
Lateral view	Talus-first metatarsal angle	±4°
	Calcaneal pitch	23–30°
	Talocalcaneal angle	35–50°

## Data Availability

Not applicable.
